# Cardiac Sarcoidosis: A Comprehensive Clinical Review

**DOI:** 10.31083/j.rcm2502037

**Published:** 2024-01-29

**Authors:** András Vereckei, Zsuzsanna Besenyi, Viktória Nagy, Bence Radics, Hajnalka Vágó, Zsigmond Jenei, Gábor Katona, Róbert Sepp

**Affiliations:** ^1^Department of Medicine and Hematology, Semmelweis University, 1088 Budapest, Hungary; ^2^Department of Nuclear Medicine, University of Szeged, 6720 Szeged, Hungary; ^3^Division of Non-Invasive Cardiology, Department of Medicine, University of Szeged, 6720 Szeged, Hungary; ^4^Department of Pathology, University of Szeged, 6720 Szeged, Hungary; ^5^Heart and Vascular Center, Semmelweis University, 1122 Budapest, Hungary

**Keywords:** sarcoidosis, cardiac sarcoidosis, granulomatous disease

## Abstract

Sarcoidosis is an inflammatory multisystemic disease of unknown etiology characterized by the formation of non-caseating granulomas. Sarcoidosis can affect any organ, predominantly the lungs, lymphatic system, skin and eyes. While >90% of patients with sarcoidosis have lung involvement, an estimated 5% of patients with sarcoidosis have clinically manifest cardiac sarcoidosis (CS), whereas approximately 25% have asymptomatic, clinically silent cardiac involvement verified by autopsy or imaging studies. CS can present with conduction disturbances, ventricular arrhythmias, heart failure or sudden cardiac death. Approximately 30% of <60-year-old patients presenting with unexplained high degree atrioventricular (AV) block or ventricular tachycardia are diagnosed with CS, therefore CS should be strongly considered in such patients. CS is the second leading cause of death among patients affected by sarcoidosis after pulmonary sarcoidosis, therefore its early recognition is important, because early treatment may prevent death from cardiovascular involvement. The establishment of isolated CS diagnosis sometimes can be quite difficult, when extracardiac disease cannot be verified. The other reason for the difficulty to diagnose CS is that CS is a chameleon of cardiology and it can mimic (completely or almost completely) different cardiac diseases, such as arrhythmogenic cardiomyopathy, giant cell myocarditis, dilated, restrictive and hypertrophic cardiomyopathies. In this review article we will discuss the current diagnosis and management of CS and delineate the potential difficulties and pitfalls of establishing the diagnosis in atypical cases of isolated CS.

## 1. Introduction

Sarcoidosis is a systemic inflammatory disease of unknown etiology characterized by multiorgan involvement and the formation of non-caseating granulomas. It is thought that exposure to certain environmental antigens (infectious, occupational or other) results in an exaggerated, dysregulated T-cell-driven immune response in patients with a genetic predisposition leading to non-necrotic granulomatous inflammation. Sarcoidosis can affect any organ, predominantly the lungs, lymphatic system, skin and eyes. While 90% of patients with sarcoidosis have lung and intrathoracic lymph node involvement, only approximately 5% have clinically manifest cardiac involvement. Another 20–25% of patients have asymptomatic, clinically silent cardiac involvement shown in autopsy studies [1, 2, 3, 4, 5, 6, 7, 8, 9]. Earlier studies [10, 11, 12] showed that most patients with clinically manifest cardiac sarcoidosis (CS) have minimal extracardiac disease and up to two thirds have isolated CS, however more recent studies [13, 14] reported a much lower 3.2% to 9.4% prevalence of isolated CS without evidence of extracardiac disease using ^18^F-fluorodeoxyglucose positron emission tomography/computed tomography (FDG-PET/CT). CS can be asymptomatic, subclinical or can present with conduction disturbances (atrioventricular block or intraventricular conduction disturbance), ventricular and atrial arrhythmias, heart failure or sudden cardiac death. Approximately 30% of <60-year-old patients presenting with unexplained high degree (Mobitz type II second degree or third degree) atrioventricular (AV) block or ventricular tachycardia (VT) are diagnosed with CS, therefore CS should be strongly considered in such patients [6, 15, 16, 17, 18]. CS is the second leading cause of death in patients with sarcoidosis after pulmonary sarcoidosis and the leading cause of death among Japanese sarcoidosis patients, therefore its early recognition is important, because its early treatment may prevent death from cardiovascular involvement [4, 8, 10]. The establishment of CS diagnosis can be quite difficult, because CS is a chameleon of cardiology able to mimic sometimes completely different cardiac diseases, such as arrhythmogenic cardiomyopathy (ACM), giant cell, lymphocytic, eosinophilic myocarditis, non-ischemic dilated cardiomyopathy, restrictive and hypertrophic cardiomyopathies [7, 9, 19, 20].

## 2. Epidemiology

The prevalence of systemic sarcoidosis is between 5 and 64 per 100,000 of the population. A higher prevalence has been reported in Scandinavian countries and among African Americans and the lowest prevalence was found among Asians [21, 22, 23, 24]. Most disease occurs in patients between 25 and 60 years of age and sarcoidosis is unusual in people under the age of 15 or older than 70 years, the disease affects both sexes, with slight predominance in women [4, 5, 8, 25].

## 3. Pathogenesis and Etiology

The inciting antigen, which might be an infectious agent, environmental antigen or an autoantigen in individuals with genetic predisposition, and/or certain human leukocyte antigen (HLA) polymorphisms trigger the formation of non-necrotizing granulomas. Antigen-presenting cells, such as macrophages and dendritic cells, process the inciting antigen and induce cell-mediated immune reaction by activating na$\ddot{\iota }$ve CD4+ T-cells, that results in the proliferation of T-helper (Th)1 and Th17 T-cells, which secrete proinflammatory cytokines, such as interleukin (IL)-2, IL-12, tumor necrosis factor (TNF)-α and interferon-γ. These cytokines aggregate macrophages, and lymphocytes into primary granulomas surrounding the inciting antigen. Macrophages then turn into epithelioid cells, fusing to form multinucleated giant cells. In the chronic phase there is a shift from Th1 to Th2-cells secreting IL-4, IL-10 and tumor growth factor (TGF)-β, which promote fibroblast recruitment and extracellular matrix deposition and fibrosis [1, 26].

Among the several infectious agents suggested to have a role in the etiology of sarcoidosis Propionibacterium acnes is the only microorganism, which was isolated from sarcoid lesions [10, 27, 28, 29]. There is a familial clustering of cases in sarcoidosis, as the first- and second-degree relatives are more affected than the general population [30]. The Case Control Etiology of Sarcoidosis Study (ACCESS) study showed that patients who are first-degree relatives of patients with sarcoidosis had a five times higher risk of developing sarcoidosis compared to controls [31].

## 4. Clinical Presentation

Isolated CS is a more serious disease than CS associated with extracardiac sarcoidosis [11, 32]. Cardiac manifestations of CS include ventricular and atrial arrhythmias, AV or intraventricular conduction disturbance, sinus node dysfunction, heart failure, sudden cardiac death (SCD) and less commonly valvular heart disease, ischemia, pericardial disease with or without pericardial effusion. The most common symptoms of CS related to these cardiac manifestations are palpitation, presyncope, syncope, breathlessness disproportionate to the extent of pulmonary involvement, angina-like chest pain, edema or cardiac arrest, sudden cardiac death as a first presentation of the disease. Approximately 20–25% of patients with CS are asymptomatic [9, 32]. The manifestations of CS mainly depend on the location and extent of granulomas and fibrosis. AV block, bundle branch block (BBB) or sinus node dysfunction can be due to granulomatous inflammation or scar tissue in regions of the conduction system (in the sinus node or basal/mid interventricular septum) or direct involvement of the coronary artery blood supply to the conduction system (sinoatrial and AV nodal arteries) by granulomas and/or scar tissue. AV block occurs in 26–67% of CS patients, BBB has an estimated prevalence of 12–61% with right BBB (RBBB) occurring more frequently than left BBB (LBBB) [32]. Ventricular arrhythmias are mostly due to late-stage scar formation and in some cases due to small ventricular aneurysm formation serving as anatomical substrate for macroreentry, but active inflammation can also cause ventricular arrhythmias by triggered activity, increased automaticity and also by reentry mechanisms. Atrial arrhythmias, such as atrial fibrillation, atrial flutter, atrial tachycardia, are more commonly caused by atrial enlargement due to systolic or diastolic ventricular dysfunction associated with heart failure, or atrial enlargement due to pulmonary sarcoidosis-related pulmonary arterial hypertension, right heart dysfunction, than by direct granulomatous involvement of the atrial myocardium. The mechanisms of atrial arrhythmias are abnormal automaticity, macroreentry and triggered activity [9, 32]. Heart failure develops as a consequence of widespread myocardial infiltration by granulomatous inflammation and fibrosis. Angina-like chest pain, acute coronary syndrome may be due to impaired coronary flow reserve from compression of the myocardial microvasculature, rarely to granulomatous coronary arteritis, or either compression or dissection of a single coronary artery [20]. Granulomas can also involve heart valves resulting in valvular insufficiency, most commonly mitral regurgitation [32].

## 5. Diagnosis of Cardiac Sarcoidosis

The diagnosis of sarcoidosis is based on the classic triad of (1) compatible clinical characteristics, (2) histological evidence of non-caseating and non-necrotizing granulomas and (3) the exclusion of other granulomatous diseases [4, 20]. There are two major pathways for the diagnosis of CS: (1) the histological pathway, (2) the clinical diagnosis pathway. The histological pathway can be applied and the diagnosis of CS established by performing endomyocardial biopsy (EMB), which reveals non-caseating granulomas with no alternative underlying cause. Or, if EMB is not attempted or negative, which cannot rule out CS, due to the patchy nature of the disease resulting in a low sensitivity (20–30%) of detection, that despite the application of imaging-, or electroanatomical mapping-guided sampling techniques can improve to modest at best, the clinical diagnosis of CS is probable and can be established, if there is histological evidence of extracardiac sarcoidosis and the simultaneous presence of one or more suggestive cardiac findings (Table 1, Ref. [5]). The histological pathway is recommended by the Heart Rhythm Society (HRS) Expert Consensus statement [33] and the World Association for Sarcoidosis and Other Granulomatous Disorders (WASOG) Guidelines [34] (Table 1). However, the 2016 Japanese Ministry of Health and Welfare guidelines [8] also allow the diagnosis of CS without a biopsy of any affected organ and render possible the clinical diagnosis pathway when in addition to the presence of certain characteristic findings suggesting cardiac involvement, certain characteristic laboratory findings are also present (Table 2, Ref. [8]).

**Table 1.** **Heart Rhythm Society Expert Consensus Recommendations on criteria for the diagnosis of cardiac sarcoidosis (2014)**.

There are 2 pathways to a diagnosis of cardiac sarcoidosis (CS):
**1. Histological diagnosis from myocardial tissue**
CS is diagnosed if an endomyocardial biopsy shows non-caseating granuloma with no alternative cause for the histological findings identified
**2. Clinical diagnosis from invasive and non-invasive studies:**
CS is probable* if
(a) There is a histological diagnosis of extra-cardiac sarcoidosis
*and*
(b) One or more of following is present:
➢ Steroid +/- immunosuppressant responsive cardiomyopathy or heart block
➢ Unexplained reduced left ventricular ejection fraction (<40%)
➢ Unexplained sustained (spontaneous or induced) ventricular tachycardia
➢ Mobitz type II, second- or third-degree heart block
➢ Patchy uptake on dedicated cardiac FDG-PET in a pattern consistent with CS
➢ Late Gadolinium Enhancement on CMR consistent with CS pattern
➢ Positive gallium uptake in a pattern consistent with CS
*and*
(c) Other causes for the cardiac manifestation(s) have been reasonably excluded

*In general, “probable involvement” is considered adequate to establish a clinical diagnosis of CS. Adapted from [5] with minor modifications. CMR, cardiac magnetic resonance; FDG-PET, 18F-fluorodeoxyglucose positron emission tomography.

**Table 2.** **Japanese Circulation Society 2016 Guideline on diagnosis of cardiac sarcoidosis**.

**1. Histological diagnosis**
CS is diagnosed when a biopsy (endomyocardial or surgical) shows non-caseating epithelioid granulomas
**2. Clinical diagnosis**
If an endomyocardial biopsy is not performed or is negative, a diagnosis is made clinically.
CS is diagnosed clinically (1) when epithelioid granulomas are found in organs other than the heart, and clinical findings strongly suggestive of cardiac involvement by CS are present; or (2) when there is evidence of pulmonary or ophthalmic sarcoidosis and there are ≥2 characteristic laboratory and imaging findings and clinical findings strongly suggestive of cardiac involvement (≥2 major or ≥1 major and ≥2 minor criteria)
**Criteria for cardiac involvement**
Clinical findings that satisfy ≥2 major or ≥1 major and ≥2 minor criteria strongly suggest CS
1. Major criteria
(a) High-grade AV block or fatal ventricular arrhythmia (VF and sustained VT)
(b) Basal thinning of the ventricular septum or abnormal ventricular wall anatomy including ventricular aneurysm, thinning of the middle or upper ventricular septum, regional ventricular wall thickening
(c) LVEF <50% or focal ventricular wall asynergy
(d) ^67^Ga citrate scintigraphy or ^18^F-FDG-PET revealing abnormally high tracer accumulation in the heart
(e) Cardiac MRI reveals LGE of the myocardium
2. Minor criteria
(a) Abnormal ECG findings: ventricular arrhythmias including NSVT, multifocal or frequent PVCs, BBB, axis deviation or abnormal Q waves
(b) Myocardial perfusion scintigraphy (SPECT) showing perfusion defects
(c) Endomyocardial biopsy showing infiltration with monocytes and moderate to severe myocardial interstitial fibrosis
**Characteristic laboratory and imaging findings in sarcoidosis**
A diagnosis of sarcoidosis is established when ≥2 of the following findings are observed:
1. High serum ACE activity or elevated serum lysozyme levels
2. High serum soluble interleukin-2 receptor levels
3. Increased tracer uptake in ^67^Ga citrate scintigraphy or ^18^F-FDG- PET
4. A high percentage of lymphocytes in BAL with a CD4/CD8 ratio of >3.5
5. Bilateral hilar lymphadenopathy
**Isolated CS diagnostic guidelines**
Isolated CS is suspected when:
1. No clinical findings are suggestive of other organ involvement than the heart
2. Absence of increased uptake in ^67^Ga or ^18^F-FDG-PET in any organs other than the heart
3. A chest CT scan reveals no shadow along the lymphatic tracts in the lungs or no hilar and mediastinal lymphadenopathy
Isolated CS is diagnosed with:
1. Histological diagnosis: endomyocardial biopsy or surgical biopsy show non-caseating epitheloid granulomas
2. Clinical diagnosis: isolated CS diagnosis is made when criteria for cardiac involvement 1(d) and ≥3 of the 1(a), (b), (c), (e) are satisfied

Adapted from [8] with modifications. AV, atrioventricular; ACE, angiotensin-converting enzyme; BAL, bronchoalveolar lavage; BBB, bundle branch block; CS, cardiac sarcoidosis; LGE, late gadolinium enhancement; LVEF, left ventricular ejection fraction; NSVT, non-sustained ventricular tachycardia; PVC, premature ventricular complex; VF, ventricular fibrillation; VT, ventricular tachycardia; ECG, electrocardiogram; MRI, magnetic resonance imaging; CT, computed tomography; ECG, electrocardiogram; SPECT, single-photon emission computed tomography; CD4, helper T lymphocytes; CD8, cytotoxic T lymphocytes; FDG-PET, 18F-fluorodeoxyglucose positron emission tomography.

### 5.1 Screening for CS

There are two scenarios when evaluation of patients for CS should be performed: (1) screening for cardiac involvement in patients with extracardiac sarcoidosis, (2) the presence of clinical signs and symptoms raising the suspicion of CS in patients without known sarcoidosis. All patients with verified extracardiac sarcoidosis should be screened for CS, irrespective whether they have or haven’t symptoms suggesting cardiac involvement, because CS is the second leading cause of mortality in patients affected by sarcoidosis. In these patients a detailed patient history, physical examination, electrocardiogram (ECG) (probably also Holter) recording and transthoracic echocardiography should be performed for initial CS assessment according to the HRS Expert Consensus statement [33, 35]. Patient history should be considered positive if significant palpitations lasting >2 weeks or unexplained presyncope/syncope is present, complete RBBB or LBBB, or Mobitz type II second degree or third degree AV block, or pathological Q waves in ≥2 leads, or sustained/nonsustained ventricular tachycardia suggest ECG positivity and unexplained left ventricular ejection fraction <40% and/or regional wall motion abnormality and/or basal ventricular thinning and/or ventricular wall aneurysm indicate positive echocardiographic findings. If one or more of patient history, ECG, echocardiography criteria are positive, further evaluation is recommended with advanced cardiac imaging (cardiac magnetic resonance [MR] and/or FDG-PET/CT), if none of them is positive, CS is unlikely, and advanced cardiac imaging is not recommended. The suspicion of CS should also emerge in younger (<60-year-old) patients without known sarcoidosis presenting with any of the above mentioned patient history, ECG or and echocardiographic alterations. Serologic biomarker positivity, such as angiotensin-converting enzyme, troponin I, brain natriuretic peptide and other less commonly used biomarker positivity may support the suspicion of CS, but are neither sufficiently sensitive nor specific. Other common potential underlying causes (mainly ischemic heart disease) should be excluded. In these patients advanced cardiac imaging with cardiac MR and FDG-PET/CT is recommended to confirm the suspicion of CS and FDG-PET/CT is also very useful to confirm or exclude extracardiac sarcoidosis. When there is no extracardiac FDG uptake and there is no evidence of skin or eye involvement, extracardiac sarcoidosis can be ruled out. In this case isolated CS is likely if the positive patient history and/or ECG and/or echocardiographic alteration(s) were confirmed by advanced cardiac imaging. But, because even suggestive advanced imaging alterations are not specific for CS, in the case of isolated CS, imaging- or electroanatomical mapping-guided EMB should be considered to establish the diagnosis [10, 15, 20, 33, 35].

### 5.2 ECG and Holter Monitoring

**Fig. 1.**
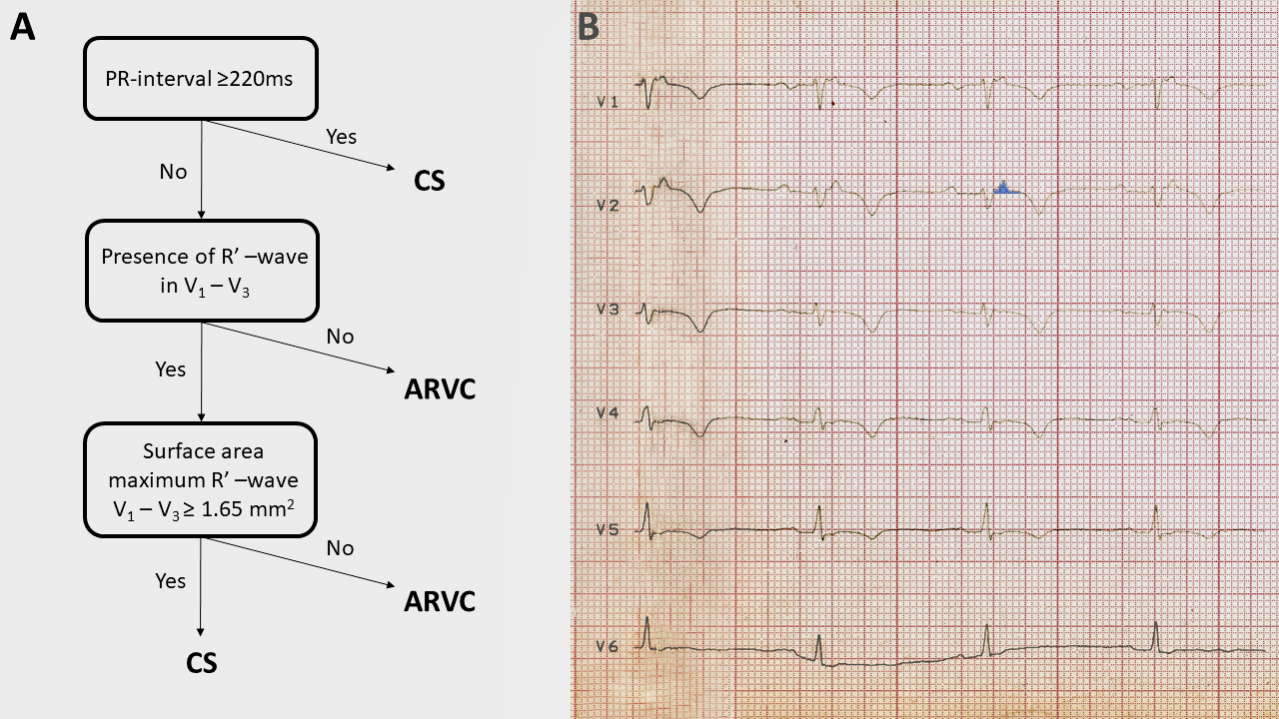
**The algorithm devised to distinguish sarcoidosis with left and right ventricular involvement from ACM and its application to the ECG of our patient who had CS mimicking ACM**. (A) The ECG algorithm. (B) The application of the algorithm on our patient’s ECG. The PR interval is 220–230 ms, thus already the first step of the algorithm suggests CS. The surface area of the maximum R’ wave in lead $V_{2}$ marked by light blue color was ≥1.65 ${\mathrm{mm}}^{2}$, therefore the third step of the algorithm also suggests CS. R’ wave was defined as any positive deflection after an S wave. Reproduced with permission from [6]. ARVC, arrhythmogenic right ventricular cardiomyopathy; CS, cardiac sarcoidosis; ECG, electrocardiogram; ACM, arrhythmogenic cardiomyopathy.

In <60-year-old patients presenting with any form of unexplained AV block, mostly high-degree (Mobitz type II second degree or third degree) AV block, intraventricular conduction disturbances [RBBB occurring more frequently than LBBB, and nonspecific intraventricular conduction disturbance (NICD)] and ventricular arrhythmias (sustained or nonsustained ventricular tachycardia, ventricular fibrillation, frequent ventricular premature beats) CS should be considered in the differential diagnosis. QRS fragmentation, as a marker of impaired conduction due to myocardial scar formation, is also more common in patients with CS, its presence together with the above mentioned ECG alterations might increase the probability of CS [16, 32, 36]. Unexplained pathological Q waves in ≥2 leads may also be present in patients with CS [10, 35]. CS may completely mimic ACM with biventricular involvement, which occurs more frequently (in 56% of patients with ACM) than the classic right ventricular (RV) dominant or isolated RV involvement form (in 39%) [37, 38], fulfilling all major non-invasive imaging and ECG criteria of ACM. CS can also be associated with T-wave inversion in right precordial leads ($V_{1-3}$) in the absence of RBBB, which is a major ECG criterion of ACM and with Ԑ-wave, which earlier was considered an almost pathognomonic ECG sign of ACM, but now is only a minor ECG criterion of ACM [6, 10, 19, 38]. Recently Hoogendoorn JC *et al*. [39] developed an ECG algorithm (Fig. 1A, Ref. [6]) including PR interval of ≥220 ms, the presence of R’ wave and the surface area of maximum R’ wave in leads $V_{1-3}$ ≥1.65 ${\mathrm{cm}}^{2}$ to distinguish CS with biventricular involvement from ACM with biventricular involvement. This algorithm worked well not only in their study, but could differentiate CS from ACM in the case of our patient [6] and on the ECG of the case report of Saturi G *et al*. [19], which are both case reports on patients with CS mimicking completely ACM (Fig. 1B). The authors do not provide a very clear explanation for the characteristic ECG alterations to CS in their algorithm, but we think that both the first degree AV block and the presence of R’ wave and the increased surface area of the R’ wave can be explained by the characteristic septal involvement in CS, which can cause AV block and impaired conduction in this area, and the latter may result in the R’ wave and its increased surface area in the right precordial leads. Atrial arrhythmia (atrial fibrillation, atrial flutter, atrial tachycardia) or sinus node dysfunction may also be present in patients with CS, but are less characteristic of CS than the aforementioned ECG alterations [32, 40].

### 5.3 Biomarkers

No pathognomonic biomarker of CS exists. Serum angiotensin converting enzyme (SACE), which is produced by activated macrophages and correlates with granuloma burden, is elevated in 30–80% of patients with active sarcoidosis, but has neither sufficient sensitivity nor specificity. SACE levels are decreased in patients treated with ACE inhibitors. Serum soluble interleukin-2 receptor (sIL-2R), which is a marker of T-cell activation, is also elevated in patients with active sarcoidosis, and can be a marker of disease activity, but it is not specific, and may be significantly elevated in other granulomatous diseases, hematological malignancies and various autoimmune disorders. Other markers of macrophage activation, such as lysozyme, neopterin, serum amyloid A, chitotriosidase may also be elevated in patients with active sarcoidosis and might be used to assess disease activity rather than as diagnostic markers, due to their low specificity. Also elevated adenosine deaminase, due to T-lymphocyte stimulation, might indicate sarcoidosis diagnosis and disease activity [1, 41, 42, 43]. Serum chitotriosidase was verified as a good biomarker of sarcoidosis, which showed a higher sensitivity and specificity than other biomarkers, and correlated well with disease activity, severity and multiorgan dissemination [44]. The presence of lymphocytosis and an increased CD4+/CD8+ cell ratio of ≥3.5 in the bronchoalveolar lavage fluid is characteristic of sarcoidosis with a sensitivity of 54–80% and a specificity of 59–80% [41]. Troponins and B-type or brain natriuretic peptide (BNP), N-terminal prohormone of brain natriuretic peptide (NT-pro-BNP) are markers of cardiac involvement in patients with sarcoidosis [1].

### 5.4 Echocardiography

Transthoracic echocardiography is usually the first imaging study performed in patients with suspected CS, and although not a sensitive and specific examination for CS, it can provide useful informations. CS can manifest with normal ventricular function or with dilated or restrictive cardiomyopathy. The most commonly observed echocardiographic abnormality is dilated cardiomyopathy with globally hypokinetic left ventricle and secondary mitral regurgitation. During an early stage of the disease thickening of the septum (usually its basal and lateral wall), sometimes with increased echogenicity, may be seen. However the thinning (<7 mm) and akinesis of the basal septum are more common, which occur in a later stage. The thinning of the basal septum, wall motion abnormalities in the absence of coronary disease and in a non-coronary distribution and the presence of ventricular aneurysm in the inferolateral wall are the relatively more specific abnormalities characteristic of CS. Left ventricular (LV) and/or RV systolic and diastolic dysfunction may be present and in end-stage disease RV dilation and dysfunction are seen. In about 20% of patients atrial wall hypertrophy may be present, rarely an appearance similar to hypertrophic cardiomyopathy can be observed. Pulmonary hypertension due to LV dysfunction, pulmonary involvement may also be present. Rarely small pericardial effusion or tamponade or constrictive pericarditis have been found. In the early stage of the disease decreased LV longitudinal function (strain), particularly in the basal interventricular septum, detected by two-dimensional (2D) speckle tracking or tissue Doppler imaging echocardiography may be present in the absence of other 2D echo alterations [1, 5, 45, 46].

### 5.5 Cardiac Magnetic Resonance (CMR)

**Fig. 2.**
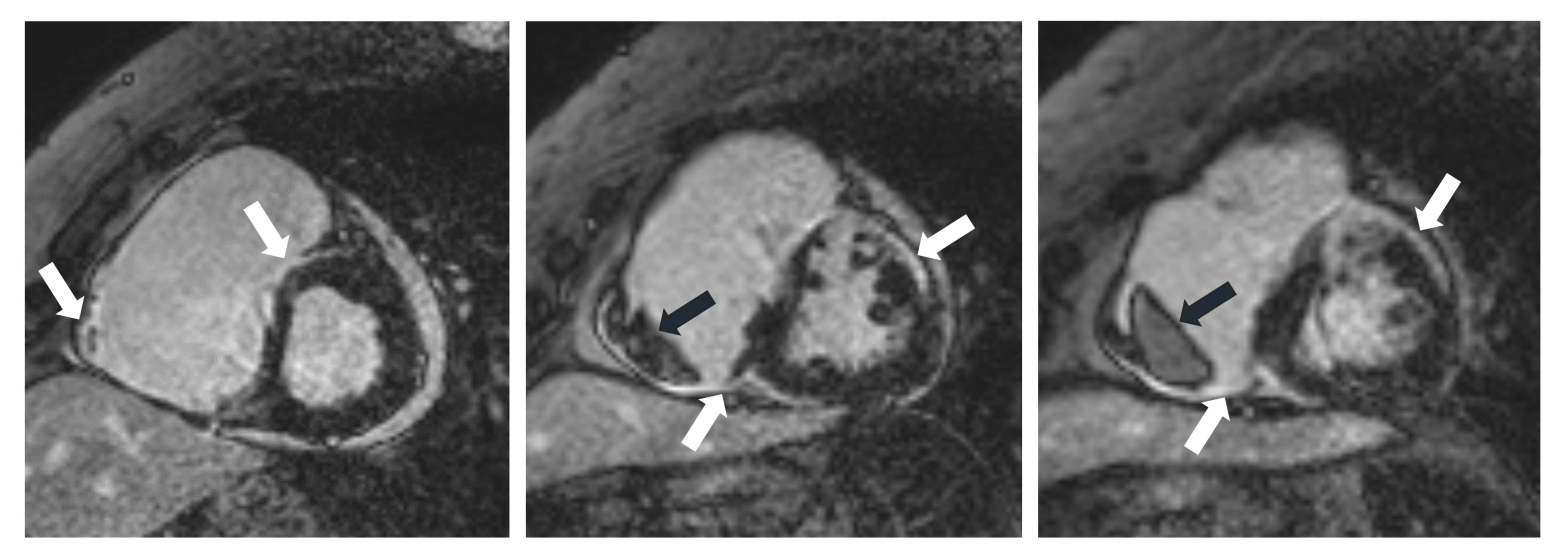
**CMR images of a 70-year-old female patient with CS**. The delayed contrast enhancement images in short axis planes show biventricular late gadolinium enhancement (LGE) corresponding to fibrotic involvement (white arrows) and right ventricular thrombus formation (black arrows). Subepicardial LGE is present in the anterior septum, LV inferior wall, subepicardial-midmyocardial LGE is seen in the LV anterior wall and LGE is present in the RV myocardium in the vicinity of thrombus. CMR, cardiac magnetic resonance; CS, cardiac sarcoidosis; LV, left ventricle; RV, right ventricle.

If the sceening tests (history, physical examination, ECG, echocardiography, biomarkers) suggest a clinical suspicion of CS, advanced imaging studies, CMR and FDG-PET/CT [usually together with resting myocardial perfusion single-photon emission computed tomography (SPECT) or positron emission tomography (PET)] are performed to confirm the presence of CS. Usually CMR is the first performed advanced imaging modality, as it is the study of choice for diagnosing cardiac involvement in sarcoidosis, due to its accuracy in the assessment of cardiac structure (capability of detecting morphological abnormalities, such as wall thinning, thickening, aneurysms) and function and tissue characterization by detection of small areas of myocardial damage due to scarring or inflammation, and its high negative predictive value (≥90%). However, CMR and FDG-PET/CT are rather complementary examinations, FDG-PET/CT in contrast to CMR, which mainly detects scar tissue and the classic fibrotic stage of CS, recognizes better the active myocardial inflammatory stage of CS, and can better guide treatment and monitor treatment response in CS than CMR, and able also to detect extra-cardiac sarcoidosis [10, 32, 35]. Typical morphological findings for CS on CMR are similar to the echocardiographic alterations and include localised myocardial thickness, basal thinning of the ventricular septum, diffuse ventricular wall thinning, ventricular dilation and ventricular aneurysm [47]. CMR can also detect myocardial edema and inflammation in CS with T2 weighted images most commonly using Short Tau Inversion Recovery (T2-STIR) methods, which are sensitive to the free water content of the tissue. Myocardial edema is represented on T2-STIR images as areas of higher signal intensity, therefore, this technique is mainly used to detect localized lesions. However, T2-STIR methods have a relatively low sensitivity due to their low contrast to noise ratio, and can also be affected by artefacts from slow-moving blood at the endocardial surface. Novel CMR T1 and T2 techniques are capable of quantitatively measuring myocardial changes. The T1 and T2 relaxation times of the myocardium can be reduced or prolonged in different conditions. Myocardial edema causes prolongation of both the T1 and T2 times, myocardial fibrosis causes prolongation of the T1 time. These changes can be objectively detected by mapping measurements even in diffuse myocardial damage [48, 49]. However, delayed contrast (15 min) imaging is the key CMR modality in CS. Late gadolinium enhancement (LGE) reflects extracellular expansion and delayed wash-out related to necrosis and edema in the acute phase and replacement fibrosis in the chronic phase. Typically subepicardial or midwall LGE in the basal and lateral LV wall and in the basal septum, distributed in a patchy, non-coronary pattern is seen. LGE in the basal anteroseptum and inferoseptum with contiguous extension into the RV free wall called as “hook sign” (or “hug sign”) indicates high probability of CS even in the absence of extracardiac biopsy evidence, but it can also be seen in giant cell myocarditis (Fig. 2). A not typical subendocardial or transmural LGE in other myocardial locations has also been described in patients with CS. The pattern of LGE in CS is non-specific and overlaps with many other pathologies. Differential diagnosis may include viral myocarditis, hypertrophic, dilated or arrhythmogenic cardiomyopathy, and coronary artery disease. In ischemic damage, the LGE progresses from the endocardial to the epicardial layer and respects a coronary territory. Dilated cardiomyopathy is characterized predominantly by linear midmyocardial LGE. In viral myocarditis, patchy subepicardial LGE can be detected particularly in the LV lateral segments. Patchy midmyocardial LGE in the hypertrophic segments is typical for hypertrophic cardiomyopathy [9, 10, 45, 50, 51, 52, 53, 54]. LGE independently predicts future adverse events, such as AV block, ventricular arrhythmias, SCD, mortality and heart failure even in patients with normal or near-normal LV ejection fraction (LVEF). A recently published meta-analysis confirmed the prognostic significance of LGE in CS. It showed that patients with known or suspected CS with LGE on CMR had a significantly higher risk for both ventricular arrhythmias and all-cause mortality. Patients with extensive LGE (>20%) have an even worse outcome than patients with a limited extent of LGE. CMR has a high diagnostic accuracy in detecting CS with a sensitivity of 75–100% and a specificity of 76–85% [9, 10, 45, 50, 55, 56, 57, 58]. However the main value of CMR in the diagnostic algorithm of CS is its high (>90%) negative predictive value. Based on a meta-analysis of 7 studies with 694 subjects, the absence of LGE has a high negative predictive value in patients with a suspicion of CS. LGE-negative patients have low incidence of cardiovascular mortality and ventricular arrhythmias [10, 59, 60]. Novel CMR T1, T2 and extracellular volume mapping techniques have incremental values in detecting subclinical CS. This technique can be useful even in subclinical CS when LGE is absent and LV systolic function is normal [61, 62, 63].

### 5.6 ^18^F-Fluorodeoxyglucose Positron Emission Tomography/Computed Tomography (FDG-PET/CT)

**Fig. 3.**
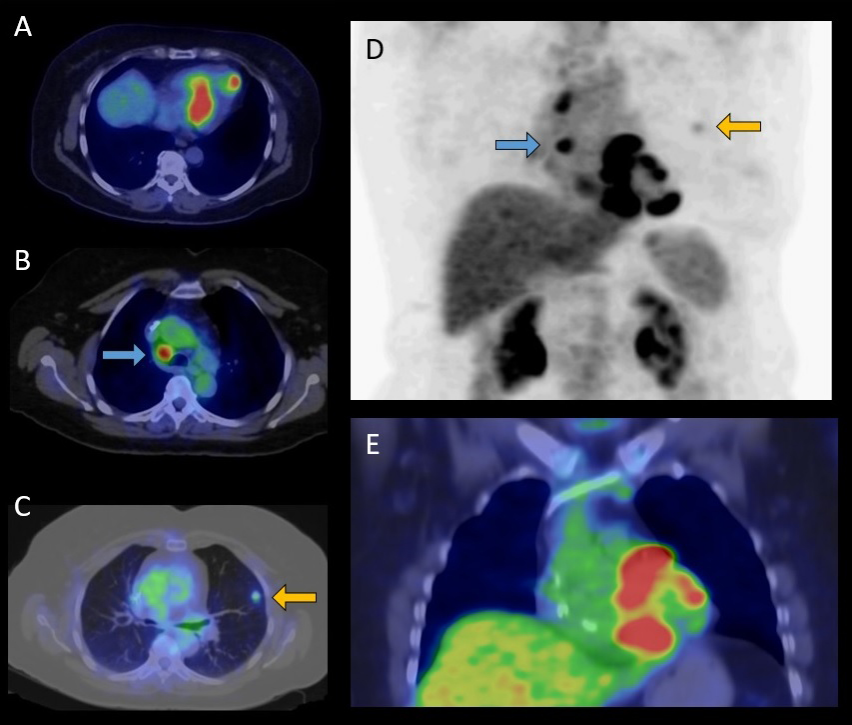
**FDG-PET/CT examination of a 65-year-old female patient with histologically (EMB) proven sarcoidosis**. Axial fused (A,B,C) and coronal fused (E) PET/CT images and maximum intensity projection (MIP) PET image (D) show increased multifocal FDG uptake in the left ventricular myocardium (A,E) as cardiac involvement, high focal lymph node uptake ((B,D) marked by blue arrows) and pulmonary uptake ((C,D) marked by yellow arrows) indicative for extracardiac manifestation of sarcoidosis. FDG-PET/CT, 18F-fluorodeoxyglucose positron emission tomography/computed tomography; EMB, endomyocardial biopsy.

Active inflammatory cells have high glycolytic activity and the accumulation of fluorodeoxyglucose (FDG) in activated macrophages and CD4+ T-lymphocytes is the underlying mechanism for *in vivo* visualization of active granulomatous sarcoid lesions. The physiologic cardiac glucose metabolism should be switched off by a low carbohydrate/high-fat diet for 12–24 h prior to the scan, followed by a 12–18 h fasting and in some centers the use of 50 IU/kg intravenous unfractionated heparin approximately 15 min prior to ^18^F-FDG injection to raise acutely free fatty acid level by activating lipoprotein and hepatic lipase, which reduces glucose consumption by the normal myocardium. The presence of “focal” or “focal on diffuse” FDG uptake is abnormal, and may be consistent with cardiac inflammation from sarcoidosis (Fig. 3). The normal FDG image pattern for an appropriately prepared patient is no myocardial FDG uptake, although low-intensity FDG uptake in the lateral wall can be a normal finding, particularly when this uptake is homogenous and not associated with any resting perfusion defects. Diffuse FDG uptake may probably indicate poor suppression of normal myocardial glucose uptake, or may represent multiple sarcoid granulomas with a diffuse distribution FDG uptake [45, 47].

A standard FDG-PET/CT is usually performed in conjunction with resting myocardial perfusion imaging to confirm the presence of CS. Both SPECT (99mTc labelled tracers) and PET (rubidium or ammonia) myocardial perfusion imaging methods are acceptable, based on the availability of different radiotracers, although the spatial resolution of PET is significantly higher compared to SPECT [64]. A “mismatch pattern” with FDG accumulation within and in the surrounding areas of a perfusion defect is highly suggestive of CS, as granulomas may impair coronary microcirculation leading to perfusion defects in non-coronary distribution, which can be reversible on treatment, but replacement fibrosis in the chronic stage causes irreversible perfusion defects, that can be associated with segmental wall motion abnormalities. The following combined FDG and myocardial perfusion imaging patterns can be present: (1) “early” (only FDG positive), (2) “progressive inflammatory” (FDG positive without major perfusion defects), (3) “peak active” (high FDG uptake with small perfusion defects), (4) “progressive myocardial impairment” (high FDG uptake with large perfusion defects), and (5) “fibrosis predominant” (perfusion defects without FDG uptake). It is mandatory to rule out coronary artery disease as an alternative diagnosis by cardiac CT or coronary angiography, if perfusion defects are present [45]. In a meta-analysis the pooled sensitivity was 89% and the specificity was 78% of FDG-PET in the detection of CS [65, 66]. In the future cardiac PET studies using tracers that work without dietary preparation, such as somatostatin analogs, and hybrid PET/CMR imaging may further improve diagnostic accuracy [20, 67]. It should be noted that abnormal FDG uptake is not specific for CS, it can also be present in ACM, Lamin A-mutation related cardiomyopathy, myocarditis (giant cell myocarditis), hibernating myocardium, connective tissue, and rheumatic disease with cardiac involvement. The absence of extracardiac uptake decreases the specificity of FDG-PET for CS [20, 47]. It is important that FDG-PET can also be used to detect extracardiac sarcoidosis. Atrial FDG uptake predicts atrial tachyarrhythmia. FDG-PET has also prognostic implications. A “mismatch pattern” and RV uptake are the key predictors of cardiac events [66, 68, 69].

### 5.7 Histological Confirmation of Sarcoidosis (Endomyocardial and Extracardiac Tissue Biopsy)

Due to the insufficient sensitivity and associated risk of endomyocardial biopsy, the diagnosis in the majority of CS cases is based on findings of extracardiac tissue biopsy combined with the patient’s clinical presentation and advanced cardiac imaging findings. Chest CT or whole-body FDG-PET scan can identify lung tissue and mediastinal or hilar lymphadenopathy suitable for extracardiac tissue biopsy. Endobronchial ultrasound-guided biopsy is preferable over mediastinoscopy for lymph node biopsy and provides a higher yield, has a better sensitivity and lower procedural risk than endomyocardial biopsy [1, 20, 35]. Due to the patchy distribution of non-caseating granulomas in CS, endomyocardial biopsy (EMB) performed as a non-targeted RV biopsy has a poor diagnostic yield and a low 20–30% sensitivity. This can be improved by performing CMR or FDG-PET or electroanatomical mapping guided EMB, if a clear involvement of the RV or the interventricular septum can be verified, and by taking more, 10–15 heart muscle samples. By using these methods the sensitivity of EMB can be increased to 50–77% [15, 33, 70, 71]. Potential complications of EMB include rupture of the RV free wall causing tamponade, conduction disturbance, arrhythmias, pneumothorax, tricuspid valve regurgitation and pulmonary embolism. The risk of complications is relatively low, <1%, when performed by an experienced physician [47].

**Fig. 4.**
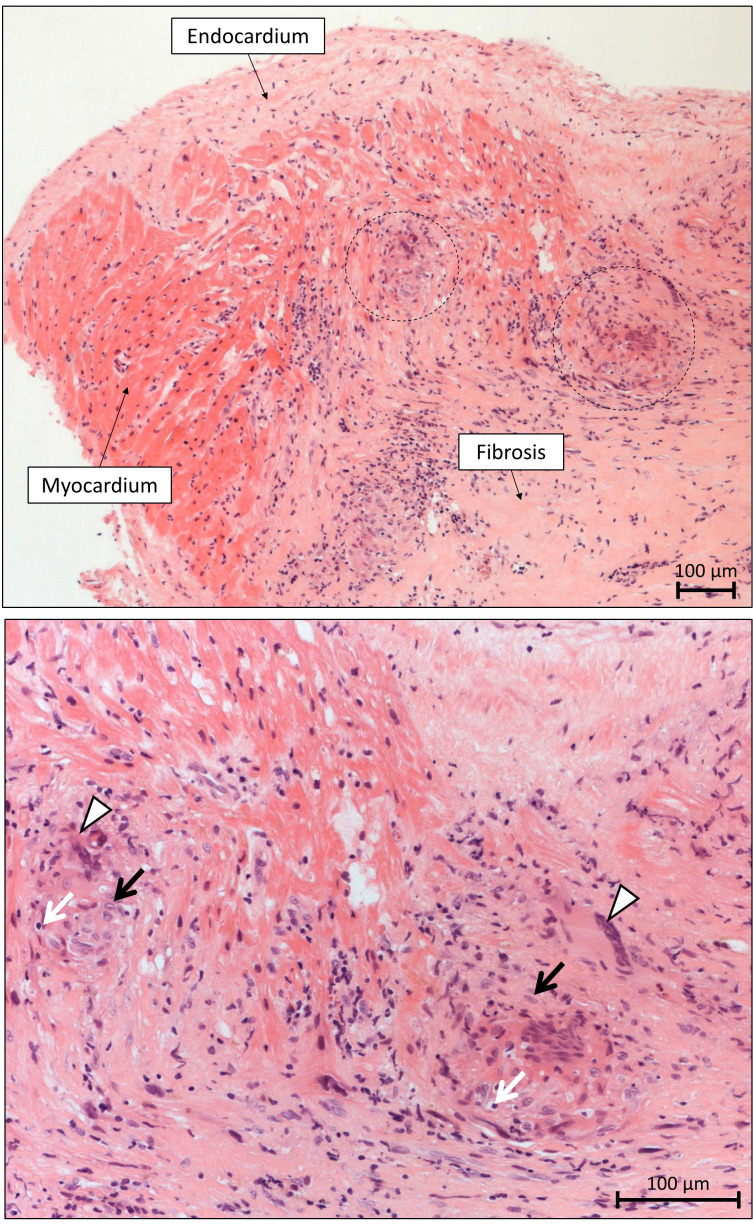
**Microscopic appearance of sarcoidosis in the endomyocardial biopsy specimen**. Top: The non-necrotizing granulomatous inflammation of the myocardium is sharply demarcated, and it is typically surrounded by diffuse fibrosis. The granulomas (- - -) are scattered and typically well circumscribed in sarcoidosis. Diffuse necrosis of cardiomyocytes is absent (H&E staining, 10(×) objective magnification). Bottom: The cellular components of sarcoid granulomas include multinucleated giant cells (Δ), epithelioid macrophages (black arrows) and lymphocytes (white arrows) (H&E staining, 20(×) objective magnification).

The hallmark histological finding in CS is non-caseating, non-necrotizing granulomas composed of aggregates of tightly clustered epithelioid macrophages often with multinucleated giant cells with or without surrounding lymphocytic/granulocytic infiltration combined with myocardial fibrosis, sharply demarcated areas of involvement, but no extensive eosinophilia or myocyte necrosis (Fig. 4). However, the typical non-caseating granulomas are seldom observed in the EMB specimen, therefore diagnostic confirmation of CS is often difficult. A combination of some novel surrogate histological findings, such as microgranulomas, increased number of dendritic cells, the accumulation of pro-inflammatory M1 (${\mathrm{CD68}}^{+}$${\mathrm{CD163}}^{-}$) macrophages and decreased number of anti-inflammatory M2 (${\mathrm{CD68}}^{+}$${\mathrm{CD163}}^{+}$) macrophages, lymphangiogenesis (increased lymphatic vessel count), confluent fibrosis and fatty infiltration, the detection of monoclonal antibody against Propionibacterium acnes by immunohistochemistry may be useful in the histological diagnosis of CS in the absence of typical non-caseating granulomas [72, 73, 74, 75]. The giant cells may contain cytoplasmic inclusions, particularly Schaumann or asteroid bodies. Schaumann bodies, which are oval, concentric laminations of calcified proteins, are often identified in multinucleated giant cells in sarcoid granulomas (up to 88% of cases versus 10% in infectious granulomatous diseases), whereas asteroid bodies, which are star-shaped structures composed of filamentous microtubular materials, are less frequently observed. These inclusion bodies in multinucleated giant cells in granulomas are non-specific for sarcoidosis [76].

## 6. Differential Diagnosis

In the differential diagnosis of CS ACM, desmoplakin cardiomyopathy, lymphocytic, eosinophilic and giant cell myocarditis, non-ischemic dilated cardiomyopathy or ischemic cardiomyopathy, restrictive cardiomyopathy, some genetic cardiomyopathies and granulomatous infections should be mostly considered [20, 26]. The difficulty in distinguishing CS from other cardiac diseases is indicated by the fact that the classification of CS is not yet fully determined. In the 2023 ESC Cardiomyopathy Guideline CS is classified as a dilated cardiomyopathy, in a review article as a restrictive cardiomyopathy, and in the 2022 ESC Guideline on ventricular arrhythmias and SCD neither as dilated nor as restrictive cardiomyopathy, but as an inflammatory cardiac disease [77, 78, 79]. CS can perfectly mimic ACM with biventricular involvement fulfilling its all diagnostic criteria, however presentation of symptoms at an older age, negative family history, AV conduction abnormalities (any degree of AV block), the presence of R’ wave and the surface area of the maximum R’ wave in leads $V_{1-3}$ ≥1.65 ${\mathrm{mm}}^{2}$, significant LV dysfunction, involvement of the ventricular septum and mediastinal lymphadenopathy should raise the suspicion of CS [6, 16, 19, 20]. Desmoplakin cardiomyopathy, a variant of ACM, which is different from the classical ACM, because it tends to disproportionally involve the LV and presents frequently with myocardial injury with chest pain and troponin elevation and associated with a worse clinical outcome, may also emerge as a possibility in the differential diagnosis [80]. The distinction of CS from giant cell myocarditis (GCM) can also be very difficult, an important reason for this fact, that they share many common characteristics and there is even a debate whether they are distinct diseases or parts of a one-disease continuum. If we consider them two distinct diseases, in clinical presentation patients with GCM compared with CS more often have acute heart failure with a rapid clinical course, significantly impaired LVEF, but less LV dilation, and much higher natriuretic peptide and troponin levels, suggesting a more intensive and acute myocardial injury, significantly worse event free survival, but this latter feature is rather due mostly to the presence of more extensive myocardial injury and more severe LV dysfunction, than the diagnosis in itself. On histopathology the presence of non-necrotizing epithelioid cell granulomas together with multinuclear giant cells, fibrosis, sharply demarcated areas of inflammation and absence of considerable myocardial necrosis and eosinophilic infiltration are suggestive of CS, whereas myocyte necrosis and eosinophilic infiltration are suggestive of GCM. In GCM granulomas are either not present, or if present, they are poorly organized. Well-organized, follicular granulomas containing central giant cells exclude the diagnosis of GCM [81, 82, 83, 84]. There are many common characteristics of CS and GCM. They are both T-cell-mediated inflammatory cardiomyopathies, both can be associated with thymomas autoimmune diseases. Septal thinning, considered a hallmark of CS, is common in GCM, patients with GCM can also show FDG-PET uptake in the heart, both diseases are more common in women, and although CS presents initially infrequently with heart failure, and if presents, it is typically subacute, sometimes it may present as a fulminant acute heart failure, similar to GCM. The histology features of GCM and CS also overlap and their distinction can be very difficult, sometimes even a matter of judgement, despite the above mentioned differences. This statement is supported by a study in which 60% of patients classified earlier as GCM by histology were reclassified as CS. The main reason for the reclassification was finding granulomas that had been missed or misinterpreted during the earlier examination [81, 85]. Several authors reported in patients with confirmed extracardiac sarcoidosis GCM in their hearts [86, 87, 88, 89]. These findings, the many common clinical and histological features, and the reclassification of many patients with histological diagnosis of GCM as CS might suggest that CS and GCM are severity phenotypes of a single disease. Advanced CS can be misdiagnosed as dilated cardiomyopathy. Several transplant centers have reported that all their cases of CS in the explanted heart had a pretransplant misdiagnosis of idiopathic dilated cardiomyopathy [20, 90, 91, 92].

## 7. Treatment

Patients with clinically manifest symptomatic CS are treated with immunosuppressive therapy. However, there is no evidence and therefore no consensus whether treatment should be started based on the presence of active lesions or based on the presence of clinical symptomatology. Whether immunosuppressive therapy should be initiated in patients with asymptomatic, metabolically active CS on FDG-PET and normal ventricular function without conduction disturbance and ventricular arrhythmias is less clear, because there is no unequivocal evidence from randomized studies for the benefit of immunosuppression in these patients. Therefore some experts recommend individualized treatment of these patients based on the consideration of the extent of myocardial inflammation, systemic involvement and potential risks of therapy. But many other experts recommend the immunosuppressive treatment of these asymptomatic patients in order to prevent disease progression to fibrosis and later severe cardiovascular complications [10, 20, 26]. Sarcoidosis experts agree on the treatment of CS with immunosuppressive therapy for the following clinical scenarios: LV dysfunction, ventricular arrhythmias, hypermetabolic activity on cardiac FDG-PET, presence of conduction defects, LGE on CMR, or RV dysfunction in the absence of pulmonary hypertension [45]. Isolated CS has a poorer prognosis than CS associated with systemic sarcoidosis, because it presents with lower LVEF and frequent ventricular arrhythmias and SCD. Therefore its treatment might be more indicated, even in asymptomatic cases [46, 93, 94].

### 7.1 Immunosuppressive Therapy

#### 7.1.1 First-Line Therapy

Immunosuppression with corticosteroids is the first-line treatment of patients with CS. A review of 34 clinical reports involving 1297 patients concluded that corticosteroids improve AV conduction in 40% of patients and may prevent the deterioration of LV function, whereas their effect on ventricular arrhythmias and mortality remains ambiguous due to poor data quality [20, 95]. There are contradictory results whether corticosteroid therapy is beneficial in patients with severe LV dysfunction (LVEF <30%), some studies reported that patients with severe LV dysfunction did not improve with treatment, other authors reported improvement of severe LV dysfunction [96, 97, 98]. The efficacy of immunosuppressive therapy probably depends on whether the cardiac manifestation of sarcoidosis is due to active inflammation or fibrosis, and the extent and proportion of inflammation and fibrosis. The usual dosage of oral prednisone is 30–40 mg/day or 0.5 mg/kg/day. In refractory or severe cases, such as rapidly progressive heart failure, life-threatening arrhythmias and extensive inflammation on cardiac PET, intravenous methylprednisolone in a dose of 500–1000 mg/day in 2–3 successive days is given or the addition of a steroid-sparing agent to a higher dose (1–1.5 mg/kg/day) of prednisone may be tried [20, 26, 47, 99]. The optimal dose, duration and tapering regimen for corticosteroid therapy have not been established. The dose of prednisone is slowly tapered to 5–15 mg/day after 1 to 3 months and the duration of corticosteroid treatment is ≥12 months (12–16 months). When initially prednisone is administered together with another immunosuppressive agent, its initial dose is ≤20 mg/day [20, 100].

#### 7.1.2 Second-Line Immunosuppressive Treatment

Second-line immunosuppressive agents, including methotrexate, azathioprine, mycophenolate mofetil, leflunomide, cyclophosphamide, are used in patients with refractory disease, or if the dose of corticosteroid needs to be reduced to prevent or diminish its adverse effects. The most commonly used second-line agent is methotrexate used in a weekly dose of 10–20 mg. Azathioprine, which is also used frequently, is applied in a 1–2 mg/kg body weight/day dose. Both need follow-up to check for adverse effects, including bone marrow suppression, infections, hepatotoxicity, renal failure, gastrointestinal complications, interstitial pneumonitis, pulmonary fibrosis, and teratogenicity. There are some data supporting combination therapy from the very beginning, but there is not yet a good evidence for improved outcome achieved by this combined therapy compared with only corticosteroid treatment. However, due to the multiple significant side effects of corticosteroid therapy, many centers consider the initial use of combined therapy with medium to low dose of corticosteroids in association with a sparing immunosuppressive agent, such as methotrexate [20, 101].

#### 7.1.3 Third-Line Treatment

Biological therapy using TNF-α inhibitors, such as infliximab or adalimumab, or lymphocyte-targeted therapy with rituximab can be beneficial in CS, when other treatments have failed. Before the start of TNF-α inhibitor therapy screening for tuberculosis and viral or any severe other infections is necessary, and TNF-α inhibitors are contraindicated in moderate to severe (New York Heart Association [NYHA] Class III–IV) congestive heart failure, multiple sclerosis or optic neuritis. Infliximab is administered in a 5 mg/kg dose at weeks 0.2 and 4 and every 8th week thereafter for one year or until signs of inflammation abate. Adalimumab in 40 mg sc. injection is administered biweekly [20, 101].

#### 7.1.4 Ongoing Trials Investigating Immunosuppressive Therapy

The Cardiac Sarcoidosis Multicenter Randomized Trial (CHASM CS-RCT) that tests the hypothesis that low-dose prednisone-methotrexate combination is as effective as a standard dose of prednisone is expected to publish the results in approximately 3 years. The Japanese Antibacterial Drug Management for CS (J-ACNES) trial is a randomized, multicenter trial comparing corticosteroid therapy given alone or together with antibiotics (chlarithromycin and doxycycline) based on the assumed pathogenetic role of Propionibacterium acnes. The Interleukin-1 Blockade for Treatment of CS (MAGIC-ART) is a randomized trial comparing standard care alone with standard care+IL-1 blocker (anakinra) treatment in CS. The RESOLVE-Heart trial is investigating the efficacy, safety and tolerability of namilumab, a monoclonal antibody, targeting the granulocyte macrophage colony stimulating factor in active CS [20, 46].

#### 7.1.5 Monitoring Response to Treatment

**Fig. 5.**
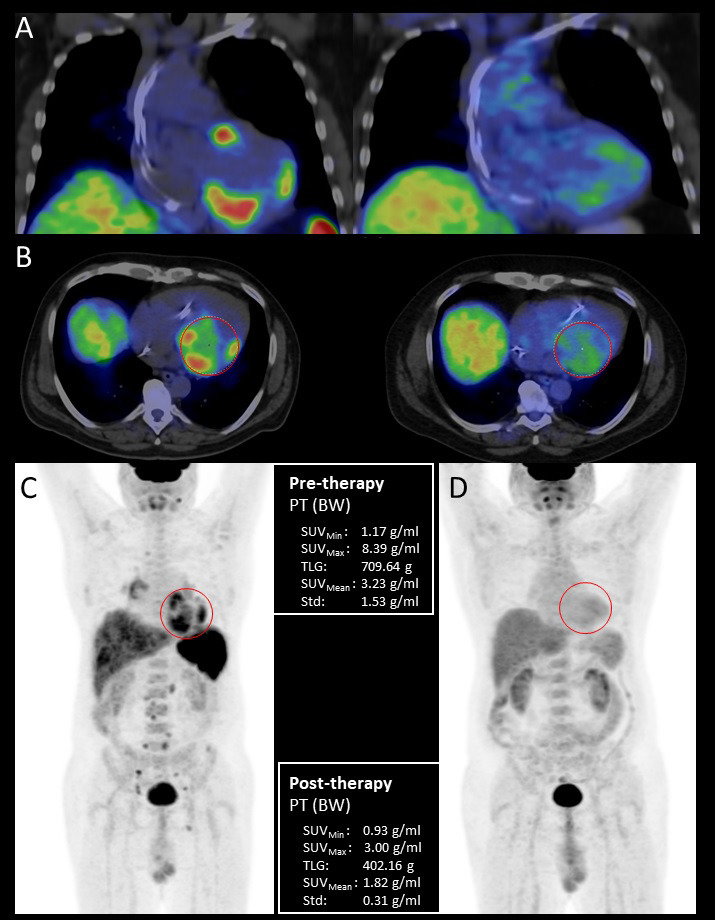
**Pre- and posttreatment FDG-PET/CT examination of a 44-year-old male patient with histologically (EMB) proven sarcoidosis**. Coronal fused pretreatment and posttreatment (A) and axial fused (B) PET/CT images with volume of interest (VOI) and maximum intensity projection (MIP) PET images before (C) and after immunosuppressive therapy (D) with quantitative parameters. Pretreatment scans show increased multifocal FDG uptake in the left and right ventricular myocardium as cardiac involvement, the presence of high focal supra- and infradiaphragmatic lymph node uptake is indicative of extracardiac sarcoidosis. Posttreatment scans do not show pathological FDG uptake confirming complete treatment response. EMB, endomyocardial biopsy; BW, body weight; FDG-PET/CT, 18F-fluorodeoxyglucose positron emission tomography/computed tomography; PT, positron emission tomography; SUV, standardized uptake values; TLG, total glycolytic activity.

In many centers repeated FDG-PET studies are performed as a gold standard test to determine the extent, presence or absence of myocardial inflammation and its response to therapy and to tailor treatment accordingly. It was shown that reduction of myocardial inflammation was associated with an improvement in LVEF [26, 46, 47, 102]. Several studies indicated that serial FDG-PET is feasible to determine the extent of disease activity and to quantitatively assess the response of CS to therapy [103, 104]. To evaluate response to treatment baseline and follow-up cardiac FDG-PET scans are performed. The therapeutic response is analyzed visually and quantitatively, the widely used quantitative parameters are the maximum and mean standardized uptake values (${\mathrm{SUV}}_{\text{max}}$, ${\mathrm{SUV}}_{\text{mean}}$) and total glycolytic activity (TLG). Fig. 5 suggests a complete treatment response. Other centers use clinical assessment, ECG, device interrogation, echocardiography and biomarkers to assess patient response and use a selective PET strategy, performing repeated PET study only if the results of the above mentioned examinations are discrepant, or raise suspicion of insufficient treatment response or relapse. Their rationale for the selective PET strategy is that in a recent study the rate of major cardiac events did not differ significantly between patients showing a complete clearance of FDG uptake vs. no response on early follow-up PET [20, 26, 104]. After discontinuing immunosuppressive therapy follow-up visits continue annually for 3–5 years and every other year thereafter [20].

### 7.2 Management and Prevention of Ventricular Arrhythmias, Sudden Cardiac Death, Conduction Disturbances and Heart Failure

#### 7.2.1 Ventricular Arrhythmias

In the case of active inflammation corticosteroid treatment is recommended for the treatment of ventricular arrhythmias together with antiarrhythmic drugs, mainly amiodarone or sotalol for VT. If medical therapy is not effective, and the ventricular arrhythmia is felt scar based, catheter ablation can be considered. In contrast to AV block, which primarily develops in CS during the acute, inflammatory phase, sustained VT more commonly develops in the advanced stage of CS, due to a scar-related substrate. VT ablation can help to control VT storm or incessant VTs, which have a relatively high incidence in CS. In cases refractory to medical and catheter ablation therapy bilateral cardiac sympathectomy may be considered [10, 20, 32, 35, 77, 105].

#### 7.2.2 Prevention of Sudden Cardiac Death

**Fig. 6.**
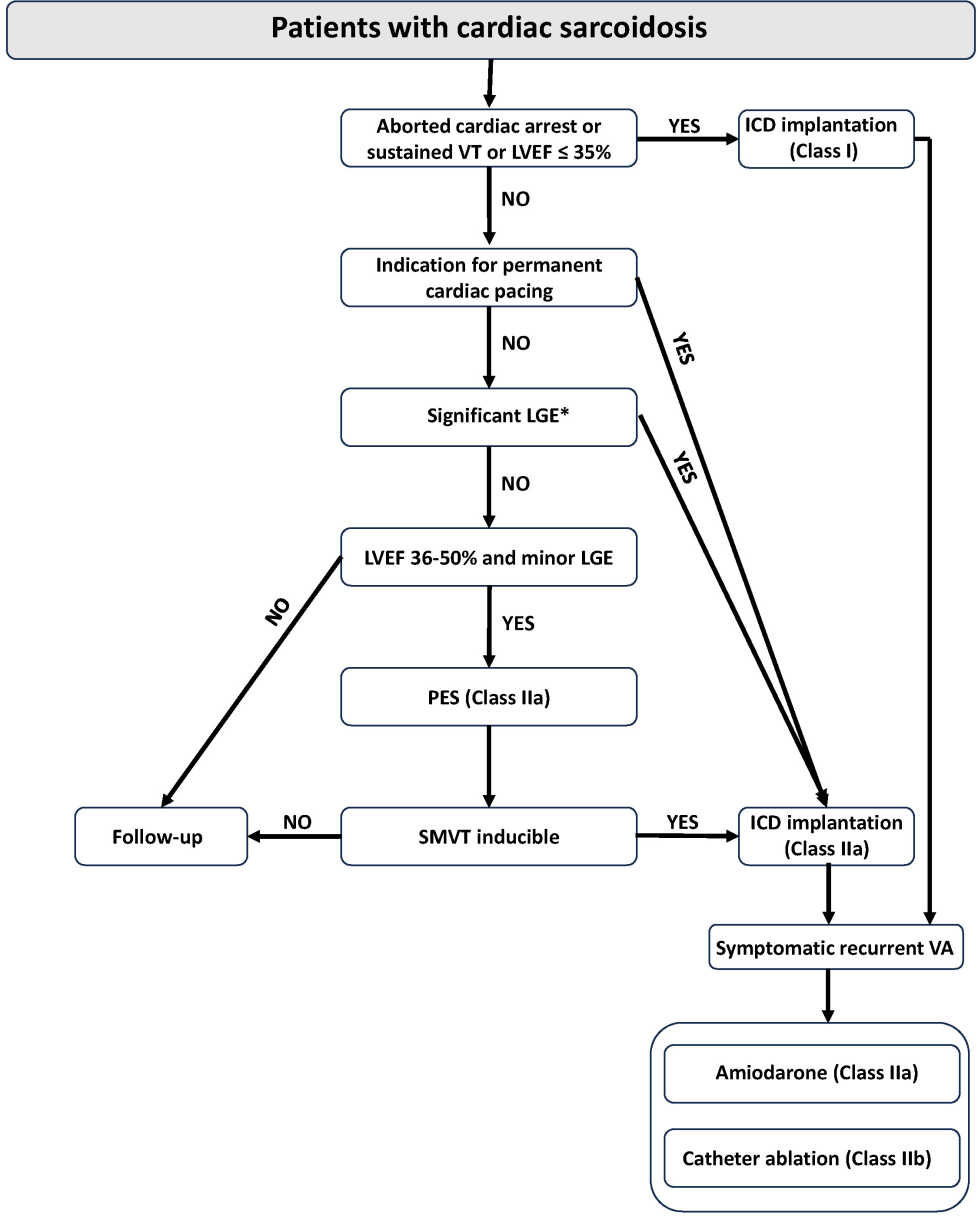
**Algorithm for sudden cardiac death prevention and treatment of ventricular arrhythmia in patients with cardiac sarcoidosis**. ICD, implantable cardioverter defibrillator; LGE, late gadolinium enhancement; LVEF, left ventricular ejection fraction; PES, programmed electrical stimulation; SMVT, sustained monomorphic ventricular tachycardia; VA, ventricular arrhythmia; VT, ventricular tachycardia. *LGE affecting ≥9/22 segments or ≥22% of the LV mass has been associated with arrhythmic endpoints. Reproduced from [79] (Fig. 24 of [79]).

SCD is considered responsible for the majority of deaths in CS, patients with clinically manifest CS having a 10% risk of SCD over 5 years of follow-up [10, 106]. Table 3 (Ref. [35]) and Fig. 6 (Ref. [79]) summarizes the recommendations given by the HRS [33], the ACC/AHA/HRS consortium [107] and the ESC [79] guidelines. General implantable cardioverter defibrillator (ICD) indications for secondary prevention, such as documented sustained VT, prior aborted cardiac arrest, are also applicable for patients with CS. In a patient with CS and LVEF ≤35% despite optimal medical therapy, ICD implantation is indicated for primary prevention of SCD. ICD implantation is also indicated in patients with CS and unexplained syncope, which is likely of arrhythmic origin. In patients with CS permanent pacing is recommended for high-degree AV block, even if the AV block improves after immunosuppressive therapy, because there is a risk of recurrent high-degree AV block. Moreover, because CS patients with AV block and preserved LV function have a 9% risk of SCD and 24% risk of SCD/VT over 5 years and these risks are even higher in CS patients with decreased LVEF or VT, it is recommended to implant an ICD in CS patients with an indication for permanent pacing, or implant a cardiac resynchronization therapy pacemaker-defibrillator (CRT-D) in patients with heart failure and intraventricular conduction disturbance with an indication for permanent pacing [10, 20, 35, 77, 108]. In CS patients with a moderately reduced LVEF (>35%) despite immunosuppressive therapy an electrophysiological study may be beneficial for further risk stratification. Programmed electric stimulation (PES) had a 75% positive predictive value and a 98.5% negative predictive value for ventricular arrhythmia in patients with subclinical CS. Therefore the induction of sustained ventricular arrhythmia during PES is an indication of ICD implantation in CS [35, 109]. A meta-analysis of 10 studies showed that the presence of LGE on CMR in CS, even in patients with preserved LV function, was associated with an increased risk of death and ventricular arrhythmias [57]. For this reason, in patients with CS with an LVEF >35% and with evidence of extensive myocardial scar, LGE or a significant LGE after resolution of acute inflammation on CMR and/or PET, ICD implantation is recommended, however a widely accepted definition of the degree of extensive or significant LGE or scarring is not yet available [20, 35]. It was earlier suggested that an LGE of ≥5% is associated with a significantly higher risk of ventricular arrhythmias and SCD [110, 111], but more recently an LGE in ≥9/29 segments (17 LV and 12 RV segments) and LGE affecting ≥22% of the LV mass have been associated with arrhythmic endpoints [77]. Several studies have shown that patients with CS with mild to moderate LV or RV systolic dysfunction despite optimal medical therapy can be at risk of arrhythmias and SCD. Therefore ICD implantation should also be considered in these patients [35, 77, 112, 113].

**Table 3.** **Indications for implantable cardioverter defibrillator (ICD) in cardiac sarcoidosis (CS)**.

**Indications for ICD in CS class of recommendation**	
Documented sustained VT, prior aborted cardiac arrest or LVEF <35% despite GDMT and immunosuppression	I
LVEF >35% with an indication for permanent pacemaker	IIa
History of syncope/near syncope compatible with arrhythmia-related etiology	IIa
Inducible sustained ventricular arrhythmia at PES	IIa
LVEF >35% with evidence of myocardial fibrosis (LGE) on CMR or PET, which is extensive or present after the resolution of acute inflammation	IIa
LVEF 36–49% and RVEF <40% despite GDMT and immunosuppression	IIb

Adapted with modifications from [35]. CS, cardiac sarcoidosis; GDMT, guideline directed medical therapy; ICD, implantable cardioverter- defibrillator; LGE, late gadolinium enhancement; PES, programmed electric stimulation; VT, ventricular tachycardia; LVEF, left ventricular ejection fraction; PET, positron emission tomography; CMR, cardiac magnetic resonance; RVEF, right ventricular ejection fraction.

#### 7.2.3 Treatment of Heart Failure

In the rare cases of CS-related fulminant myocarditis aggressive immunosuppressive therapy and mechanical circulatory support may be necessary. In CS patients with heart failure and ventricular dyssynchrony rather CRT-D, than cardiac resynchronization therapy with a-pacemaker (CRT-P) is recommended. In patients with end-stage CS mechanical circulatory support (LV assist device) and cardiac transplantation can be considered. Patients with CS have a similar post-transplant survival and risk of late complications as other transplant recipients. Recurrence of CS in the allograft is rare and not resulted in transplant failure [20, 114, 115, 116].

## 8. Prognosis

The prognosis of CS patients is less favorable than the prognosis of patients with sarcoidosis without cardiac involvement [5]. Contemporary data show improved prognosis of patients with CS compared with earlier data, due to modern heart failure management and an increasing use of ICDs. A Finnish study of biopsy verified CS patients found a 92.5% transplant-free 10-year survivals and other studies showed ≥90% overall 5-year survivals [5, 44, 106, 113, 117]. Cardiac death in patients with CS is due to the progression of cardiac dysfunction or fatal arrhythmias leading to SCD. In patients with CS the extent of LV myocardial involvement is the most important predictor of survival indicated by LVEF, LV global longitudinal strain, quantity of LGE on CMR and segments with perfusion-metabolism mismatch. The presence of high-degree AV block, persistent myocardial inflammation, abnormal pulmonary function tests are also associated with worse prognosis. RV involvement, indicated by RVEF and LGE in the RV, was independently associated with an increased risk of mortality, SCD and ventricular arrhythmias. A clinical presentation with sustained VT or heart failure is associated with a poor prognosis, while lone AV block has a less ominous prognosis [1, 20, 97, 118].

## 9. Conclusions

Although CS is increasingly recognized, it remains a diagnostic and therapeutic challenge requiring a multidisciplinary approach. Its timely recognition and treatment has utmost importance since it is the second most frequent cause of death from sarcoidosis, and its early treatment may prevent life-threatening arrhythmias, SCD and heart failure. CS should be considered in all patients with extracardiac sarcoidosis, even if they have no symptoms suggesting cardiac involvement, and in all <60-year-old patients presenting with unexplained conduction disturbance, ventricular arrhythmia and heart failure. Advanced cardiac imaging methods (CMR and FDG-PET) facilitated the diagnosis and prognostication of CS and the assessment of the response to treatment. However, the diagnosis of isolated, subclinical CS remains very difficult. The optimal dosage, duration and drug combinations of immunosuppressive treatment needs to be determined. Most patients with clinically manifest CS require ICD implantation. There are still many unanswered questions and areas of management that need to be improved, such as the pathogenesis of sarcoidosis, the management of isolated, subclinical CS, whether they should be treated or watchful waiting is safe and can be recommended, how can we better identify patients predisposed to life-threatening arrhythmias and SCD, and how can we better determine who needs ICD implantation.
